# Fatty acid and melatonin-enriched warming: A novel approach using vitrified oocytes and early-stage embryos for patients with poor prognosis

**DOI:** 10.1371/journal.pone.0346886

**Published:** 2026-04-21

**Authors:** Yoo Ra Ko, Soyoung Kim, Jinkyeong Ha, Seyul Han, Woo Sik Lee, Jin Hee Eum

**Affiliations:** 1 Department of Obstetrics and Gynaecology, CHA University Fertility Center, Gangnam, Seoul, Republic of Korea; 2 IVF and Fertility Research Laboratory, CHA University Fertility Center, Gangnam, Seoul, Republic of Korea; 3 Seoul Fertility Clinic, Seoul, Republic of Korea; Cardiff Metropolitan University, UNITED KINGDOM OF GREAT BRITAIN AND NORTHERN IRELAND

## Abstract

**Background:**

Cryopreservation and warming can induce cellular stress that impairs oocyte and embryo competence, particularly in poor-prognosis patients. Although fatty acids and melatonin have been proposed to support cytoskeletal stability and reduce oxidative stress, their combined use during warming has not been specifically evaluated. This study evaluated whether co-supplementation with fatty acids and melatonin during warming improves embryological development and clinical outcomes following thawing of vitrified oocytes or early-stage embryos.

**Methods:**

This retrospective cohort study was conducted at a single fertility centre between August 2022 and December 2024. The embryo-thawed cohort comprised women aged ≥35 years with poor embryo quality, defined as failure to achieve blastocyst cryopreservation in at least two prior *in vitro* fertilisation cycles. The oocyte-thawed cohort included women aged ≥35 years at vitrification. Warming was performed with or without 1% fatty acids and 10 µM melatonin. Embryological and clinical outcomes were compared using non-parametric analyses, with age-adjusted comparisons by multivariable regression.

**Results:**

In the oocyte-thawed cohort, warming with fatty acids and melatonin significantly improved oocyte survival (91.4% vs. 76.7%), spindle alignment (71.2% vs. 55.1%), and embryo development, with higher good-quality cleavage (51.0% vs. 24.1%) and blastocyst formation rates (24.6% vs. 10.3%) (all *P* < 0.001). Clinical pregnancy was also higher in the supplemented group (38.5% vs. 16.1%; adjusted OR 3.21; 95% CI, 1.17–8.82; *P* = 0.024). In the embryo-thawed cohort, enriched warming increased blastocyst formation (41.4% vs. 29.3%; *P* = 0.024), high-quality blastocyst rate (51.1% vs. 27.3%; *P* < 0.001), implantation (26.1% vs. 14.4%; *P* = 0.002), and clinical pregnancy (51.4% vs. 29.1%; adjusted OR 3.13; 95% CI, 1.75–5.60; *P* < 0.001).

**Conclusions:**

Co-supplementation with fatty acids and melatonin during warming improved oocyte survival, spindle alignment, embryo development, and clinical pregnancy outcomes in poor-prognosis patients. This simple, clinically applicable approach may reduce cryo-induced stress and enhance reproductive success.

## Introduction

The transition from slow freezing to vitrification has enabled the widespread adoption of frozen–thawed embryo transfer (FET) and oocyte cryopreservation [1,2]. FET improves clinical outcomes, reduces the risk of ovarian hyperstimulation syndrome, and enables the use of preimplantation genetic testing [3,4]. Moreover, the 2020 guideline of the European Society of Human Reproduction and Embryology (ESHRE) recognised oocyte vitrification for fertility preservation as a non-experimental procedure, underscoring its clinical significance [5].

Despite these advancements, the vitrification-warming process can compromise oocyte and embryo development by disrupting cytoskeletal integrity, altering lipid composition, and inducing oxidative stress [6–12]. These adverse effects are partly mediated by cryoprotectants such as dimethyl sulfoxide (DMSO). Biophysical studies have shown that DMSO interacts with lipid bilayers and alters membrane organisation, leading to increased membrane disorder and transient changes in permeability [13–14]. Consistent with this, cryobiological studies have associated DMSO exposure with altered membrane permeability and downstream developmental outcomes [15–17]. Such transient membrane alterations may provide a limited opportunity for solution-derived molecules to access intracellular compartments during the brief warming phase.

Given this potential, melatonin (MEL) and fatty acids (FA) have been studied for their distinct mechanisms in protecting gametes and embryos during cryopreservation. MEL, which is present in follicular and tubal fluids, exerts antioxidant and anti-apoptotic effects during gamete maturation and early embryonic development [18]. In most studies, its use has been examined in culture media [19–22]; however, recent evidence suggests that supplementation during warming may reduce oxidative stress, preserve mitochondrial function, maintain oolemma permeability, and ultimately improve embryo quality and clinical outcomes [23–24]. Complementing the antioxidant action of MEL, FA facilitates mitochondrial β-oxidation, promoting adenosine triphosphate (ATP) production and cellular homeostasis during cryopreservation [25–27]. During preimplantation development, embryos undergo metabolic reprogramming toward oxidative phosphorylation, with lipid droplet remodelling and unsaturated FA biosynthesis enhancing membrane fluidity and polarity establishment [28–33].

Recent studies have shown that FA supplementation during warming improves blastocyst quality and clinical outcomes by restoring lipid droplet content, enhancing β-oxidation, and reducing apoptosis [34–37]. Although modifications to warming solutions using MEL or FA individually have been examined in human oocytes and embryos [23,24,34–37], their combined co-supplementation during warming has not been investigated. This combined approach may be particularly beneficial for women of advanced maternal age, who are vulnerable to cryo-induced damage from mitochondrial dysfunction and oxidative stress [38,39]. In addition to age-related susceptibility, thawed oocytes and early-stage embryos exhibit greater vulnerability as a result of their limited DNA repair capacity, low mitochondrial reserves, and lack of protective structures [40, 41].

Given these biological vulnerabilities, we hypothesised that targeted modification of the warming solution could improve embryological and clinical outcomes in women of advanced maternal age with compromised developmental potential. As the combined use of FA and MEL during warming has not previously been investigated in this setting, we evaluated its impact on post-thaw survival, embryo development, and clinical pregnancy outcomes.

## Materials and methods

### Patient selection and study design

This retrospective cohort study included women who underwent warming of vitrified oocytes or cleavage- and morula-stage embryos between August 2022 and December 2024 at CHA University Fertility Center, Gangnam. For research purposes, investigators accessed identifiable clinical records between 22/04/2025 and 31/05/2025; however, all data were fully de-identified immediately following the extraction process. The embryo-thawed cohort included women aged ≥35 years with recurrent poor embryo quality, defined as failure to achieve blastocyst cryopreservation in ≥2 prior *in vitro* fertilisation (IVF) cycles despite extended culture. The oocyte-thawed cohort comprised women aged ≥35 years at the time of oocyte vitrification. In both cohorts, women were excluded if they had endometriosis; obesity, defined as a body mass index (BMI) >25 kg/m² according to the 2022 guidelines of the Korean Society for the Study of Obesity [42]; autoimmune disease; or metabolic disorders such as hyperlipidaemia, hypertension, or diabetes mellitus. Each cohort was further classified based on whether FA and MEL (FA + MEL) supplementation was used during warming.

The thawed embryo cohort comprised 228 women, with 659 thawed embryos (338 and 321 in the control and FA + MEL groups, respectively). In this cohort, vitrified embryos had originally been generated using either conventional IVF or intracytoplasmic sperm injection (ICSI), according to semen parameters. Specifically, ICSI was performed in all cases of confirmed male factor infertility, in accordance with standard clinical practice. All patients in this cohort proceeded to embryo transfer (ET), with 250 embryos transferred in the control group and 249 in the FA + MEL group. Cycles were excluded if the embryos were of poor quality at the time of vitrification, defined by the presence of >50% of blastomeres with large vacuoles (>14 μm), severe fragmentation (>35%), or multinucleation. Embryos with significantly delayed development—such as day 3 embryos with only 2–4 cells—were also excluded, as these factors could confound post-warming culture outcomes [43].

**Fig 1:**
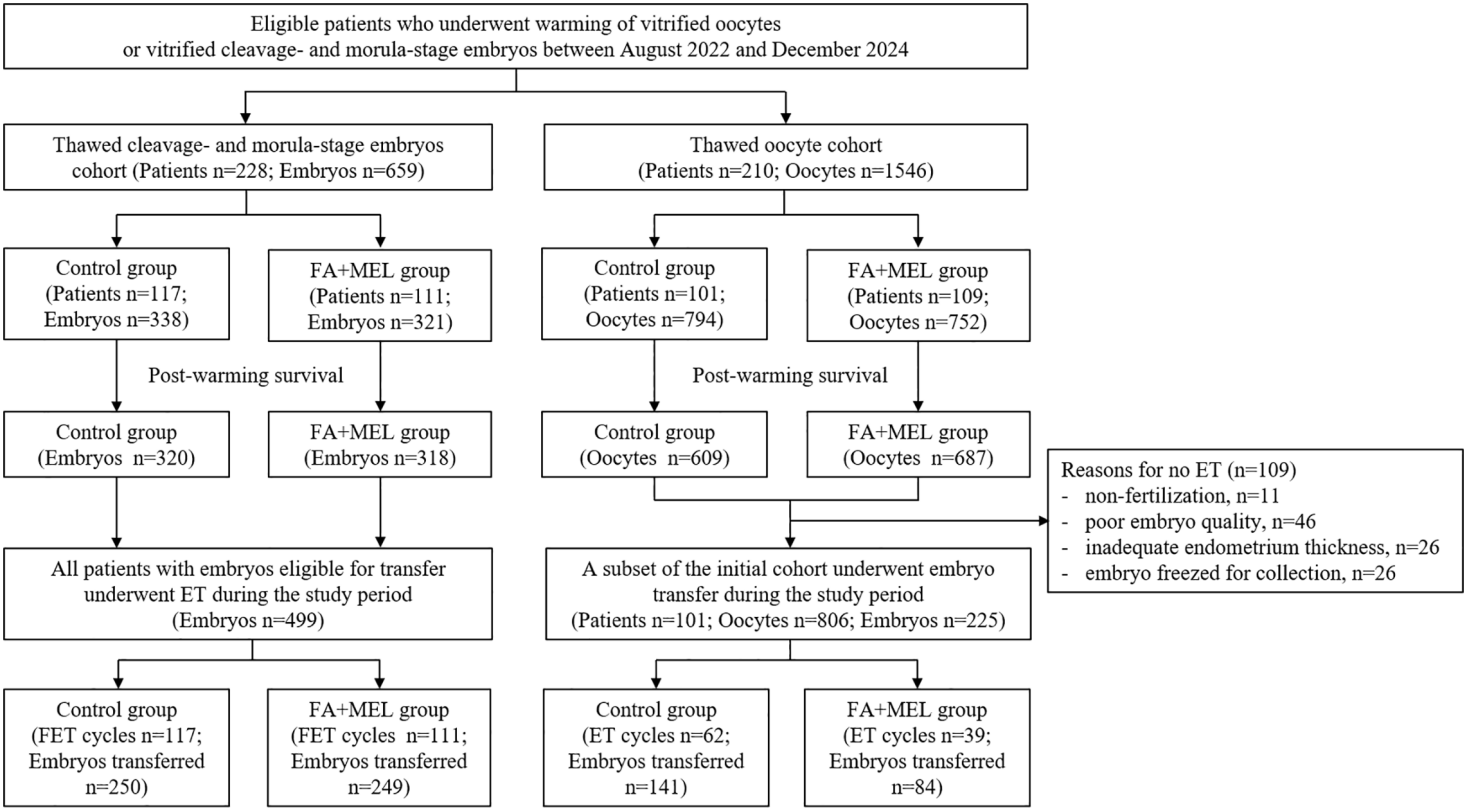
Flowchart of study participants in the thawed-embryo and -oocyte cohorts. FA, fatty acids; MEL, melatonin; FET, frozen-thawed embryo transfer; ET, embryo transfer.

The thawed oocyte cohort included 210 women, with 794 and 752 oocytes thawed in the control and FA + MEL groups, respectively. To standardise fertilisation conditions, all surviving mature oocytes underwent fertilisation via ICSI. As a result of non-fertilisation (n = 11), poor embryo quality precluding transfer (n = 46), insufficient endometrial thickness (n = 26), or elective re-cryopreservation for embryo accumulation (n = 26), the final ET cohort comprised 62 patients in the control group and 39 in the FA + MEL group. The proportion of exclusion because of poor embryo quality was comparable between the groups (control: 23/609, 3.8% vs. FA + MEL: 23/686, 3.4%; *P* = 0.79), indicating no imbalance in attrition attributable to early developmental arrest. These groups used 141 and 84 embryos, respectively, derived from the thawed oocytes (Fig 1).

This study was approved by the Institutional Review Board of CHA University Fertility Center, Gangnam (IRB approval number: 2025-03-007). As this was a retrospective analysis of anonymised clinical data, the requirement for informed written or verbal consent was waived in accordance with institutional and national guidelines.

### Oocyte and embryo vitrification

Vitrification of mature oocytes and cleavage- or morula-stage embryos was performed using a two-step protocol based on ethylene glycol (EG; Sigma-Aldrich, St. Louis, MO, USA; Cat. No. 102466), DMSO (Sigma-Aldrich; Cat. No. D2650), and sucrose (Sigma-Aldrich; Cat. No. S1888), with SAGE Quinn’s Advantage Medium with HEPES (CooperSurgical, Trumbull, CT, USA) as the base medium. To ensure consistent quality, all vitrification solutions were freshly prepared under standardised laboratory conditions. All procedures were conducted at 25 °C for oocytes and at 37 °C for embryos. Equilibration was performed under strictly timed conditions in an equilibrium solution consisting of 7.5% EG and 7.5% DMSO in HEPES medium supplemented with 20% human serum albumin (HSA). Equilibration was carried out for 7.5 min for oocytes at room temperature and for 2.5 min for cleavage- and morula-stage embryos at 37 °C. Subsequently, the samples were transferred to a vitrification solution containing 15% EG, 15% DMSO, and 0.5 M sucrose for 1 min (oocytes) and 20 s (embryos). After equilibration, samples were loaded onto gold electron microscopy grids (Gilder, Westchester, PA, USA) and rapidly plunged into liquid nitrogen for vitrification.

### Warming of oocytes and embryos with FA+ MEL-supplemented media

Vitrified oocytes and cleavage- or morula-stage embryos were warmed using a stepwise sucrose dilution protocol in HEPES-buffered medium supplemented with 20% HSA (CooperSurgical, Trumbull, CT, USA). For cleavage- and morula-stage embryos, a four-step dilution protocol was used (0.5 M, 0.25 M, 0.125 M, and 0.0 M sucrose), with 2.5 min of exposure at each step. For oocytes, a five-step protocol was applied (1.0 M, 0.5 M, 0.25 M, 0.125 M, and 0.0 M sucrose), also with 2.5 min of exposure at each step. In the FA+ MEL group, all warming solutions were supplemented with 1% FA (Gibco, Thermo Fisher Scientific, Waltham, MA, USA; Cat. No. 11905-031) and 10 μM MEL (Sigma-Aldrich, St. Louis, MO, USA; Cat. No. M5250). These supplemented warming solutions were freshly prepared under standardised laboratory conditions. Prior to clinical use, each batch underwent quality control, including verification of osmolarity and pH within predefined acceptable ranges. All solutions were prepared using sterile techniques, passed through 0.22 μm filters, and used within a specified time frame according to laboratory standard operating procedures.

**Fig 2:**
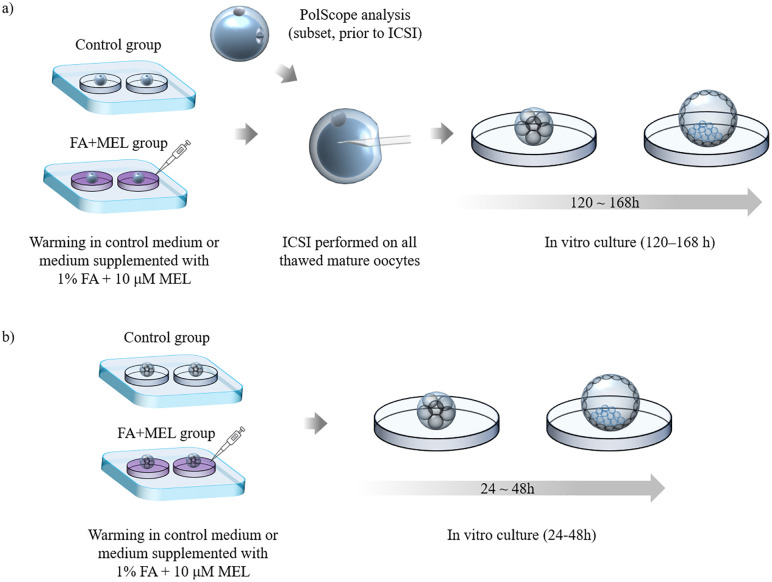
Schematic overview of the experimental workflow following vitrified-warming of oocytes and embryos. (a) Mature oocytes were warmed in control medium or medium supplemented with 1% fatty acids and 10 μM MEL, followed by ICSI. A subset was assessed for meiotic spindle angle using PolScope before ICSI. Resulting embryos were cultured for 120–168 h to evaluate developmental progression. (b) Cleavage- and morula-stage embryos were warmed in control or FA+ MEL-supplemented medium and cultured for 24–48 h to assess further development. FA, fatty acids; MEL, melatonin; ICSI, intracytoplasmic sperm injection.

Following warming, mature oocytes underwent ICSI under standard conditions. Cleavage-and morula-stage embryos were cultured in SAGE Quinn’s Advantage Blastocyst Medium under reduced oxygen (5% O_2_, 6% CO_2_, 37 °C) using the oil-drop culture method in Oosafe four-well dishes. Cleavage-stage embryos were cultured for 2 additional days before transfer, whereas morula-stage embryos were cultured overnight. Post-warming survival was defined as the presence of 100% viable blastomeres (Fig 2).

### Controlled ovarian stimulation and ET protocol

All patients underwent controlled ovarian stimulation using either gonadotropin-releasing hormone (GnRH) agonist or antagonist protocols for pituitary suppression. Stimulation regimens were individualised based on patient age, ovarian reserve markers, and previous responses to gonadotropins. Ovulation was triggered with 250–500 μg recombinant human chorionic gonadotropin (Ovidrel®, Serono, Modugno, Italy) combined with 0.1–0.2 mg GnRH agonist (Decapeptyl®, Ferring, Sweden) when at least three follicles ≥17 mm or two follicles ≥18 mm in diameter were observed. Oocyte retrieval was performed 34–36 h after trigger administration.

Endometrial preparation for FET, including ET using previously cryopreserved oocytes, was performed using one of the following protocols: (i) hormone replacement therapy (HRT) with oral oestradiol valerate 2 mg thrice daily (Progynova®, Merck, Poland), initiated on day 2 or 3 of the menstrual cycle; (ii) a modified natural cycle using letrozole 5 mg daily (Femara®, Novartis, Switzerland), also starting on day 2 or 3; or (iii) a true natural cycle.

In HRT cycles, the timing of ET was determined by endometrial thickness and the initiation of luteal phase support. In modified and natural cycles, transfer timing was based on the estimated day of ovulation. The specific transfer day (Day 3, 4, or 5) was individualised through multidisciplinary consultation between clinicians and embryologists, integrating prior IVF cycle outcomes with embryological development. Given the cohort’s history of recurrent developmental arrest, a ‘rescue transfer’ strategy was adopted: embryos showing slow progression were transferred at the cleavage or morula stage, whereas those with robust potential were cultured to the blastocyst stage. Personalised luteal phase support was provided in all thawed ET cycles. Given the patients’ history of poor clinical outcomes, a maximum of two blastocysts or three cleavage- or morula-stage embryos were transferred following warming.

### Embryological outcomes

Normal fertilisation was confirmed by the presence of two pronuclei and a second polar body 16–17 h after insemination, in accordance with the updated 2025 ESHRE/Alpha Scientists in Reproductive Medicine (ALPHA) Istanbul Consensus [44]. In oocyte-thawing cycles, the fertilisation rate was calculated as the proportion of surviving oocytes that underwent normal fertilisation.

Meiotic spindle angle assessment was performed in a subgroup of the thawed oocyte cohort before ICSI using the PolScope spindle view system (Oosight^TM^ META Imaging System; CRI, Woburn, MA, USA) in conjunction with glass-bottom culture dishes (MatTek, Ashland, MA, USA). The orientation of the meiotic spindle relative to the first polar body has been used as a non-invasive marker of oocyte integrity and developmental competence [45]. In this study, oocytes with meiotic spindle angles <30° were classified as normal, whereas those with angles >30° or not visible were classified as abnormal.

Embryo quality was assessed using established morphological criteria. To minimise subjective bias, two senior embryologists (each with >15 years of experience) performed all evaluations following standardised laboratory protocols based on the Istanbul Consensus. Regular inter-observer calibration was conducted to ensure scoring consistency. For cleavage-stage embryos, assessment included cell number, degree of fragmentation, and blastomere symmetry; embryos with ≥6 cells and <20% fragmentation were classified as good quality [46,47]. Morula-stage embryos were evaluated based on the degree and completeness of compaction, developmental timing, and absence of abnormalities. Good-quality morula-stage embryos were defined as those exhibiting full or near-complete compaction involving most of the embryo volume, with minimal fragmentation and appropriate developmental timing [44]. Blastocyst-stage embryos were assessed using the Gardner and Schoolcraft grading system [48,49], which evaluates the degree of blastocoel expansion, inner cell mass (ICM), and trophectoderm (TE) morphology. Blastocysts graded AA, AB, BA, or BB were classified as morphologically good quality [47,50].

### Clinical outcome assessment

Clinical outcomes included implantation and clinical pregnancy rates. Implantation was confirmed by the presence of a gestational sac on transvaginal ultrasonography at 5–6 weeks of gestation. The implantation rate was calculated as the number of gestational sacs observed divided by the number of embryos transferred. Clinical pregnancy was defined as the presence of foetal cardiac activity confirmed by transvaginal ultrasonography at 7–8 weeks of gestation, and the clinical pregnancy rate was calculated per ET cycle.

### Statistical analysis

Data distribution normality was assessed using the Shapiro–Wilk test, which revealed non-normal distributions; therefore, non-parametric tests were applied. Continuous variables are expressed as the mean ± standard error of the mean and were compared using the Mann–Whitney U test. Categorical variables are presented as percentages and compared using the Chi-square test. Univariate and multivariate regression analyses identified age as the only significant covariate. Accordingly, clinical outcome comparisons between groups were adjusted for age, using linear and multivariate logistic regression for continuous and categorical variables, respectively. Because embryo stage at transfer in the thawed-oocyte cohort represents post-warming developmental progression, it was not included as a baseline covariate in the primary models to avoid over-adjustment of a variable lying on the causal pathway. All statistical analyses were conducted using IBM SPSS Statistics version 29.0 (IBM Corp., Armonk, NY, USA). A *P*-value < 0.05 was considered statistically significant.

## Results

### Participant characteristics

In the thawed embryo cohort, the control group comprised 117 patients and 338 embryos, whereas the FA + MEL group included 111 patients and 321 embryos. Both groups comprised women of advanced maternal age, with mean ages of 41.0 ± 0.32 and 41.7 ± 0.32 years in the control and FA + MEL groups, respectively. The mean anti-Müllerian hormone (AMH) level was equally low in both groups (1.6 ng/mL). All patients had undergone multiple IVF and ET cycles. No significant between-group differences were observed in baseline variables (Table 1).

**Table 1:** Baseline characteristics of patients in the control and FA + MEL groups within the thawed-embryo cohort.

Characteristics	Control (n = 117)	FA + MEL (n = 111)	*P*-value
Age (years)	41.0 ± 0.32	41.7 ± 0.32	0.124
BMI (kg/m²)	21.0 ± 0.20	20.9 ± 0.19	0.574
Duration of infertility (years)	4.0 ± 0.36	3.6 ± 0.36	0.158
Number of previous IVF cycles	5.6 ± 0.28	5.3 ± 0.34	0.149
Number of previous embryo transfer cycles	2.5 ± 0.19	2.3 ± 0.14	0.784
Baseline FSH (mIU/mL)	8.4 ± 0.66	8.8 ± 0.54	0.074
AMH (ng/mL)	1.6 ± 0.17	1.6 ± 0.15	0.301
Endometrial thickness at transfer (mm)	8.8 ± 0.14	9.2 ± 0.18	0.114
Endometrial preparation, n (%)			0.246
Natural cycle	73 (62.4)	64 (57.7)	0.096
Modified natural cycle	5 (4.3)	11 (9.9)	0.466
HRT cycle	39 (33.3)	36 (32.4)	0.885
Cause of infertility, n (%)			
Male factor	11 (9.4)	18 (16.2)	0.123
Uterine factor	26 (22.2)	18 (16.2)	0.251
Unexplained	3 (2.6)	7 (6.3)	0.168
DOR	10 (8.5)	6 (5.4)	0.353
PCOS	1 (0.9)	1 (0.9)	0.825
Mixed factor	66 (56.4)	61 (55.0)	0.970

BMI, body mass index; AMH, anti-Müllerian hormone; FSH, follicle-stimulating hormone; n, number; HRT, hormone replacement therapy; DOR, diminished ovarian reserve; PCOS, polycystic ovarian syndrome; IVF, *in vitro* fertilisation; SEM, standard error of the mean; FA, fatty acids; MEL, melatonin. Mixed factor refers to patients with ≥2 infertility factors (for example, male + uterine, or DOR + unexplained). Values are presented as the mean ± SEM.

In the thawed oocyte cohort, the control group included 101 patients with 794 oocytes, and the FA + MEL group included 109 patients with 752 oocytes. The mean ages at the time of oocyte vitrification were 39.5 ± 0.42 and 38.8 ± 0.44 years in the control and FA + MEL groups, respectively. Other baseline characteristics, including BMI, AMH level, infertility duration, and history of prior IVF/ET cycles, were comparable between groups, with no significant differences (Table 2).

**Table 2:** Baseline characteristics of patients in the control and FA + MEL groups within the thawed-oocyte cohort.

Characteristics	Control (n = 101)	FA + MEL (n = 109)	*P-*value
Age at oocyte vitrification (years)	39.5 ± 0.42	38.8 ± 0.44	0.664
Age at oocyte warming (years)	41.0 ± 0.39	41.3 ± 0.40	0.639
BMI (kg/m²)	20.8 ± 0.19	20.7 ± 0.24	0.573
Duration of infertility, years	2.5 ± 0.27	2.9 ± 0.42	0.859
Number of previous IVF cycles	3.3 ± 0.31	3.4 ± 0.27	0.190
Number of previous embryo transfer cycles	1.6 ± 0.21	1.5 ± 0.18	0.652
Baseline FSH (mIU/mL)	10.3 ± 0.57	11.8 ± 1.11	0.874
AMH (ng/mL)	2.2 ± 0.29	1.5 ± 0.26	0.068
Endometrial thickness at transfer (mm)	8.8 ± 0.19	8.8 ± 0.25	0.533
Cause of infertility, n (%)			
Male factor	18 (17.8)	13 (11.9)	0.229
Uterine factor	22 (21.8)	23 (21.1)	0.904
Unexplained	6 (5.9)	8 (7.3)	0.685
DOR	8 (7.9)	10 (9.2)	0.746
PCOS	6 (5.9)	4 (3.7)	0.440
Mixed factor	41 (40.6)	51 (46.8)	0.366

BMI, body mass index; AMH, anti-Müllerian hormone; FSH, follicle-stimulating hormone; n, number; DOR, diminished ovarian reserve; PCOS, polycystic ovarian syndrome; IVF, *in vitro* fertilisation; SEM, standard error of the mean; FA, fatty acids; MEL, melatonin. Mixed factor refers to patients with ≥2 infertility factors (for example, male + uterine, or DOR + unexplained). Values are presented as the mean ± SEM.

Among these patients, ET following oocyte warming was performed in a subset. Baseline characteristics in this subgroup were generally comparable between groups, except for endometrial thickness, which was slightly lower in the FA + MEL group but remained within clinically acceptable limits. Multivariate regression analysis showed that endometrial thickness was not a significant predictor of implantation or clinical pregnancy in this subgroup (S1 Table).

### Embryological outcomes of vitrified-warmed embryos

The baseline distribution of developmental stage (cleavage-stage vs. morula-stage) and morphological quality prior to warming was comparable between the control and FA + MEL groups (*P* > 0.05). Most embryos in both groups were cryopreserved at the cleavage stage. Following warming, the FA + MEL group demonstrated a significantly higher blastocyst formation rate than the control group, with blastocysts forming in 41.4% of embryos compared with 29.3% in controls (*P* = 0.024). The proportion of high-quality blastocysts was also significantly higher in the FA + MEL group than in the control group (51.1% vs. 27.3%; *P* < 0.001).

ICM morphological analysis revealed a significantly greater proportion of grade A embryos and fewer grade C embryos in the FA + MEL group than in the control group. TE grading showed a similar trend, with more embryos classified as grade B and fewer as grade C in the FA + MEL group. The FA + MEL group had a slightly higher proportion of grade A TE embryos; however, the difference was not statistically significant (Table 3).

**Table 3:** Pre- and post-warming embryo characteristics and developmental outcomes in the control and FA + MEL groups within the thawed-embryo cohort.

Outcomes	Control (n = 117)	FA + MEL (n = 111)	*P*-value
Number of embryos	338	321	
Post-warming embryo survival rate, n (%)	320 (94.7)	318 (99.1)	0.709
Embryo stage and morphologic quality before warming, n (%)			
Morula stage embryos	115 (34.0)	106 (33.0)	0.621
High-quality morula stage embryos	51 (15.1)	49 (15.3)	0.511
Cleavage-stage embryos	223 (66.0)	215 (67.0)	0.621
High-quality cleavage stage embryos	88 (26.0)	79 (24.6)	0.595
Embryo development into blastocysts after warming, n/N (%)			
Blastocyst formation rate	99/338 (29.3)	133/321 (41.4)	0.024
High-quality blastocyst rate	27/99 (27.3)	68/133 (51.1)	< 0.001
ICM morphology grade, n/N (%)			
Grade A	5/99 (5.1)	22/133 (16.5)	0.007
Grade B	66/99 (66.7)	97/133 (72.9)	0.302
Grade C	28/99 (28.3)	14/133 (10.5)	< 0.001
TE morphology grade, n/N (%)			
Grade A	6/99 (6.1)	14/133 (10.5)	0.231
Grade B	26/99 (26.3)	56/133 (42.1)	0.013
Grade C	67/99 (67.7)	63/133 (47.4)	0.002

ICM, inner cell mass; TE, trophectoderm; n, number; N, total number of embryos; FA, fatty acids; MEL, melatonin. n/N indicates the number of blastocysts or morphological grades per total embryos thawed or blastocysts formed.

### Clinical outcomes of the vitrified-warmed embryos

In total, 250 and 249 embryos were transferred in the control and FA + MEL groups, respectively, with an average of approximately 2 embryos per cycle in both groups. The distribution of embryo developmental stages at transfer was comparable between groups (*P* > 0.05), confirming that the clinical decision-making process for transfer timing was applied consistently across both cohorts. Notably, blastocyst-stage transfers were more frequent in the FA + MEL group; however, the difference was not statistically significant. The FA + MEL group showed a significantly higher implantation rate (26.1%) than the control group (14.4%) (*P* = 0.003). Similarly, the clinical pregnancy rate was significantly improved (51.4% vs. 29.1%; *P* < 0.001). After adjusting for maternal age, both outcomes remained significant: the implantation rate increased by 13.2% in the FA + MEL group (95% confidence interval [CI]: 4.84–21.57; *P* = 0.002), and the odds of clinical pregnancy were more than 3-fold higher (adjusted odds ratio [OR]: 3.13; 95% CI: 1.75–5.60; *P* < 0.001) (Table 4).

**Table 4:** Clinical outcomes of patients in the control and FA + MEL groups within the thawed-embryo cohort.

Outcomes	Control (n = 117)	FA + MEL (n = 111)	*P*-value	Adjusted Effect (95% CI)	Adjusted *P*-value
Total number of embryos transferred	250	249			
Embryos transferred per cycle (mean ± SEM)	2.1 ± 0.08	2.2 ± 0.06	0.063	–	
Embryo stage at transfer, n/N (%)					
Cleavage-stage	15/117 (12.8)	11/111 (9.9)	0.490	–	
Morula stage	35/117 (29.9)	28/111 (25.2)	0.429	–	
Blastocyst stage	67/117 (57.3)	72/111 (64.9)	0.240	–	
Implantation rate, n/N (%)	36/250 (14.4)	65/249 (26.1)	0.003	β = 13.21 (95% CI: 4.84–21.57)	0.002
Clinical pregnancy rate, n/N (%)	34/117 (29.1)	57/111 (51.4)	< 0.001	OR: 3.13 (95% CI: 1.75–5.60)	< 0.001

CI, confidence interval; SEM, standard error of the mean; n, number; ET, embryo transfer; FA, fatty acids; MEL, melatonin. n/N values indicate ETs performed at each developmental stage per total transfer cycles; implantation events per embryos transferred; and clinical pregnancies per ET cycle. No adjustment was applied where no correlation with maternal age was found. Adjustment for maternal age was calculated using linear or logistic regression, depending on the outcome type.

### Embryological outcomes of vitrified-warmed oocytes

The post-warming oocyte survival rate was significantly higher in the FA + MEL group than in the control group (91.4% vs. 76.7%; *P* < 0.001). Fertilisation and abnormal fertilisation rates did not differ significantly between groups; however, FA + MEL supplementation significantly improved embryo development following fertilisation. The proportion of embryos reaching the six-cell stage increased from 31.8% to 72.6% (*P* < 0.001), whereas the high-quality cleavage rate (24.1% vs. 51.0%; *P* < 0.001) and blastocyst formation rate (10.3% vs. 24.6%; *P* < 0.001) were significantly higher in the FA + MEL group than in the control group. The improvement in the high-quality blastocyst rate was more modest (25.0% vs. 33.9%; *P* = 0.045); however, maternal age was identified as a confounding factor for this outcome. After adjustment for age, the FA + MEL group still demonstrated a significantly higher rate, with an estimated difference of 30.3% (95% CI: 5.42–55.16, *P* = 0.018) (Table 5).

**Table 5:** Post-warming survival, fertilisation, and embryo development from vitrified-warmed oocytes in the control and FA + MEL groups within the thawed-oocyte cohort.

Outcomes	Control (n = 101)	FA + MEL (n = 109)	*P-*value	Adjusted Effect (95% CI)	Adjusted *P-*value
Number of oocytes	794	752			
Post-warming oocyte survival, n (%)	609 (76.7)	687 (91.4)	< 0.001	–	
Number of oocytes inseminated by ICSI	609	687			
Fertilisation rate, n (%)	390 (64.0)	467 (68.0)	0.615	–	
Abnormal fertilisation rate, n (%)	37 (6.1)	32 (4.7)	0.314	–	
Embryo development after warming, n/N (%)					
Six-cell stage rate	124/390 (31.8)	339/467 (72.6)	< 0.001	–	
High-quality cleavage rate	94/390 (24.1)	238/467 (51.0)	< 0.001	–	
Blastocyst formation rate	40/390 (10.3)	115/467 (24.6)	< 0.001	–	
High-quality blastocyst rate	10/40 (25.0)	39/115 (33.9)	0.045	β = 30.29 (95% CI: 5.42–55.16)	0.018

n, number; N, total number of fertilised oocytes; ICSI, intracytoplasmic sperm injection; CI, confidence interval; FA, fatty acids; MEL, melatonin. n/N indicates the number of embryos or blastocysts reaching a specified developmental stage per normally fertilised oocyte (2PN). Abnormal fertilisation was defined as 1PN (monopronuclear) or 3PN (tripronuclear) zygotes. No adjustment was applied where no correlation with maternal age was observed. Adjustment for maternal age was calculated using linear or logistic regression, depending on the outcome type.

### Spindle localisation in thawed oocytes

In a patient subset, meiotic spindle localisation was assessed using PolScope imaging prior to ICSI. Overall, 930 metaphase II oocytes were analysed: 374 and 556 from the control and FA + MEL groups, respectively. The proportion of oocytes with normal spindle localisation was significantly higher in the FA + MEL group than in the control group, accompanied by a corresponding decrease in abnormal localisation (*P* < 0.001 and *P* = 0.049, respectively) (Table 6).

**Table 6:** Meiotic spindle localisation in thawed MII oocytes from the control and FA + MEL groups within a subset of the thawed-oocyte cohort.

	Control (n = 58)	FA + MEL (n = 90)	*P*-value
Number of MII oocytes	374	556	
Normal localisation, n (%)	206 (55.1)	396 (71.2)	< 0.001
Abnormal localisation, n (%)	168 (44.9)	160 (28.8)	0.049

MII, metaphase II; n, number.

### Clinical ET outcomes using vitrified-warmed oocytes

Both groups had a mean of approximately two embryos transferred per cycle. However, the distribution of developmental stages at the time of transfer differed significantly between the groups. In the FA + MEL group, cleavage-stage transfers were significantly less frequent (48.7% vs. 83.9%, *P* < 0.001), whereas morula-stage (25.6% vs. 6.5%) and blastocyst-stage (25.6% vs. 9.7%) transfers were numerically higher but did not reach statistical significance. The FA + MEL group also demonstrated significantly higher implantation (20.2% vs. 8.5%, *P* = 0.016) and clinical pregnancy (38.5% vs. 16.1%, *P* = 0.011) rates than the control group. Among these outcomes, only clinical pregnancy was significantly associated with maternal age. After adjustment, the FA + MEL group still exhibited a significantly higher clinical pregnancy rate, with an adjusted OR of 3.21 (95% CI: 1.17–8.82; *P* = 0.024) (Table 7).

**Table 7:** Clinical outcomes of patients in the control and FA + MEL groups that underwent embryo transfer with vitrified-warmed oocytes.

Outcomes	Control (n = 62)	FA + MEL (n = 39)	*P*-value	Adjusted Effect (95% CI)	Adjusted *P*-value
Number of oocytes	509	297			
Post-warming oocyte survival, n (%) Number of survived oocytes	390 (76.6)	266 (89.6)	0.017	–	
Number of oocytes inseminated by ICSI	390	266			
Fertilisation rate, n (%)	255 (65.4)	178 (66.9)	0.391	–	
Total number of transferred embryos	141	84			
Embryos transferred per cycle (mean ± SEM)	2.1 ± 0.09	2.0 ± 0.14	0.729	–	
Endometrial preparation, n (%)			0.297		
Natural cycle	31 (50.0)	24 (61.5)	0.165		
Modified natural cycle	17 (27.4)	7 (17.9)	0.160		
HRT cycle	14 (22.6)	8 (20.5)	0.806		
Embryo stage at transfer, n (%)					
Cleavage-stage	52 (83.9)	19 (48.7)	< 0.001	–	
Morula stage	4 (6.5)	10 (25.6)	0.007	–	
Blastocyst stage	6 (9.7)	10 (25.6)	0.032	–	
Implantation rate, n/N (%)	12 (8.5)	17 (20.2)	0.016	–	
Clinical pregnancy rate, n/N (%)	10 (16.1)	15 (38.5)	0.011	OR = 3.21 (95% CI, 1.17–8.82)	0.024

CI, confidence interval; SEM, standard error of the mean; n, number; ICSI, intracytoplasmic sperm injection; FA, fatty acids; MEL, melatonin; HRT, hormone replacement therapy. No adjustment was applied where no correlation with maternal age was found. Adjustment for maternal age was calculated using linear or logistic regression, depending on the outcome type.

## Discussion

With the rise in maternal age and the expanding use of oocyte and embryo vitrification, optimising post-thaw survival and developmental outcomes in patients with poor prognosis remains a major challenge in assisted reproductive technology. In this study, FA + MEL co-supplementation during warming enhanced embryological and clinical outcomes, suggesting a promising approach to mitigate cryo-induced damage in this population.

In the oocyte-warming cohort, FA + MEL supplementation improved post-warming survival, meiotic spindle localisation, progression to the six-cell stage, which has been associated with subsequent developmental competence [47], and the proportion of high-quality six-cell embryos. Across the oocyte- and embryo-warming cohorts, blastocyst formation and high-quality blastocyst rates were higher, accompanied by corresponding increases in implantation and clinical pregnancy rates. After adjustment for maternal age, the improvement in clinical pregnancy remained statistically significant in both cohorts.

Among these outcomes, the improvement in oocyte survival was particularly noteworthy. The FA + MEL group achieved a significantly higher post-warming oocyte survival rate than the control group (91.4% vs. 76.7%, *P* < 0.001), exceeding the previously reported survival range of 81–85% in women aged >35 years [51,52]. During the same study period, the clinic-wide post-warming survival rate for autologous oocytes in our centre was 83.9%, consistent with age-matched published data [51,52,74,75].

Mechanistically, two complementary actions likely underlie this effect. First, unsaturated FA (e.g., oleic acid) enhances membrane fluidity and helps to preserve membrane integrity, thereby promoting cryosurvival [53,54]. Second, in addition to its well-established antioxidant activity in aged oocytes [55–57], MEL supplementation during vitrification–warming inhibits early apoptosis and preserves oocyte ultrastructure [23]. In mouse models, MEL treatment increases the proportion of morphologically normal vitrified oocytes [22]. Together, these structural and antioxidant effects of FA and MEL may explain the superior post-warming survival observed with FA + MEL.

Beyond survival, FA + MEL supplementation was associated with a higher proportion of oocytes exhibiting normal meiotic spindle localisation and fewer abnormalities, despite age-related susceptibility. The meiotic spindle is highly susceptible to cold-induced depolymerisation, and cryopreservation-related oxidative stress can further disrupt the spindle architecture and the cytoskeleton [58]. With advancing maternal age, increased lipid peroxidation impairs histone assembly and promotes aneuploidy [59]. Previous studies have indicated that MEL mitigates reactive oxygen species (ROS)-induced spindle damage [60], whereas conjugated linoleic acid improves spindle morphology and developmental competence under thermal stress [61]. Overall, our data support a spindle-preserving effect of FA + MEL during the warming process.

Consistent with these structural benefits, improved post-warming survival and spindle integrity were associated with enhanced oocyte developmental competence. In the vitrified-warmed oocyte cohort, the FA + MEL group exhibited higher six-cell progression and blastocyst formation rates, along with overall improvements in embryo quality. In the thawed-embryo cohort, the FA + MEL group also demonstrated higher rates of blastocyst formation and high-quality blastocysts. However, when blastocysts were stratified by ICM and TE grades, the groups did not significantly differ in the oocyte cohort (S2 Table), whereas the embryo cohort showed a significant shift toward higher ICM and TE grades. Collectively, these findings suggest that supplementation is beneficial as early as the oocyte stage, with more pronounced effects after cleavage, when ICM and TE lineages are specified.

These findings align with the heightened ATP and membrane biosynthesis demands during post-warming repair, as well as with the predominant reliance of early embryonic development on mitochondrial FA β-oxidation under limited glycolytic capacity [25,27,62]. Vitrification–warming depletes intracellular lipid droplets and damages membranes and mitochondria, thereby impairing β-oxidation and amplifying ROS generation [6–9,11].

Accordingly, exogenous FA administered during warming provides readily oxidisable substrates, replenishes lipid droplets, and supplies precursors for membrane biogenesis [36]. In parallel, MEL stabilises mitochondria, directly scavenges ROS, and upregulates antioxidant enzymes, thereby restoring redox balance and facilitating efficient β-oxidation [63,64]. Beyond its antioxidant role, MEL promotes FA metabolism via SIRT1–AMPK activation and SIRT3-mediated deacetylation of long-chain acyl-CoA dehydrogenase, enhancing β-oxidation and ATP generation [64–67]. In porcine oocytes, MEL remodels lipid droplets and upregulates β-oxidation, mitochondrial biogenesis, and developmental competence [68]. Complementarily, oleic acid activates SIRT-linked metabolic signalling [66,69], and FA supplementation during warming upregulates β-oxidation-related genes at the cleavage stage [36]. Collectively, FA + MEL co-supplementation during warming likely enhances early embryonic development and quality by providing readily oxidisable substrates and membrane precursors, stabilising mitochondrial redox status, and supporting β-oxidation.

Notably, although previous studies using FA supplementation alone did not observe enhanced blastocyst formation [34,36,37], our findings showed a significant improvement with FA + MEL treatment. The results show that MEL acts synergistically with FA to promote embryonic development and blastocyst quality [70,71].

Given that blastocyst morphology is a key determinant of implantation potential and subsequent development [48,72], these improvements likely contributed to the higher implantation and clinical pregnancy rates observed in both cohorts. Importantly, the advancement in embryo stage at transfer, particularly in the oocyte cohort, should not be interpreted as a confounding imbalance, but rather as a downstream consequence of enhanced developmental competence. By mitigating early arrest, FA + MEL supplementation enabled a greater proportion of embryos to reach the morula or blastocyst stage, thereby linking the laboratory intervention to improved clinical outcomes.

Previous studies of MEL or FA supplementation alone have shown limited clinical benefits in older patients. For example, although MEL improves oocyte survival, fertilisation, and embryo quality in young donor cycles, it does not enhance clinical outcomes [24]. Similarly, FA supplementation during embryo warming has been associated with improved blastocyst quality and higher implantation and pregnancy rates; however, these effects were primarily observed in women under 40 years of age [34,35,37].

In contrast, our findings revealed that FA + MEL co-supplementation provides significant benefits even in older patients with poor prognosis, suggesting a synergistic effect that may overcome the age-related limitations reported in previous studies. Notably, the mean age of the oocyte cohort was 39 years—an age associated with increased cryo-vulnerability and reduced efficacy of oocyte cryopreservation— highlighting the clinical relevance of these improvements [51,73–76].

To our knowledge, this is the first study that evaluated FA + MEL co-supplementation during the warming of vitrified oocytes and early-stage embryos. A major strength of this study is its focus on a clinically challenging population—women of advanced maternal age with recurrent IVF failure and poor blastocyst development—who often have limited therapeutic options and are particularly susceptible to cryopreservation-related damage. We observed consistent improvements across key outcomes, including oocyte survival and spindle integrity in the thawed-oocyte cohort, as well as embryo development and clinical outcomes in both cohorts.

Furthermore, although the timing of ET (Day 3, 4, or 5) varied, these decisions were individualised through multidisciplinary consultations based on each patient’s previous IVF history and embryological progression. This approach reflects real-world clinical practice in patients with poor prognosis, demonstrating that the observed benefits of FA + MEL are applicable to challenging clinical scenarios in which a uniform blastocyst-only policy may not be feasible. The internal validity of our findings is further strengthened by the balanced distribution of paternal factors; the prevalence of male factor infertility was comparable between groups, and the use of ICSI when indicated effectively minimised sperm-related variability at fertilisation. Finally, the relatively large cohort size within a single-centre setting, combined with standardised laboratory and clinical protocols, further enhances internal validity. Importantly, institutional blastocyst survival rates during the same period exceeded the ≥ 95% key performance indicator defined by the ESHRE Vienna consensus [77], supporting the robustness of our laboratory cryopreservation protocols.

Nevertheless, this study has some limitations. First, the relatively small sample size in the thawed-oocyte cohort may limit the precision of stage-specific estimates, although consistent directional effects were observed. Second, comprehensive mechanistic analyses—including lipid profiling, lipid droplet dynamics, intracellular ROS measurements, and direct quantification of transmembrane permeation—were not performed. Although intracellular uptake of FA and MEL during the short warming phase was not directly measured, the proposed mechanisms are consistent with their lipophilic properties and prior evidence of cryoprotectant-induced membrane alterations during warming [13–17]. Third, because this study was conducted using a DMSO-containing vitrification protocol, the generalisability of these findings to DMSO-free systems (e.g., propanediol-based protocols) remains uncertain and warrants further investigation. Fourth, embryo quality was assessed solely by static morphology rather than morphokinetic parameters, and heterogeneity in transfer timing and protocols is inherent to retrospective research. Although such variability may introduce complexity in interpretation, our data suggest that it reflects differences in developmental progression rather than systematic bias. Despite these limitations, our findings provide consistent evidence for the beneficial effects of FA + MEL co-supplementation during warming, warranting confirmation in prospective studies with mechanistic investigations.

## Conclusions

Co-supplementation with FA and MEL during the warming of vitrified oocytes and early-stage embryos significantly improves post-thaw survival, spindle integrity, embryonic development, and clinical pregnancy outcomes in women of advanced maternal age with poor prognosis. These results suggest a synergistic effect that surpasses the benefit of either supplement alone, supporting the idea that targeted enhancement of membrane stability, redox balance, and mitochondrial metabolism can meaningfully improve developmental competence after warming. This simple, low-cost modification to existing warming protocols has substantial clinical relevance for patients with limited treatment options. Prospective studies incorporating mechanistic analyses are needed to validate these findings and further elucidate how FA and MEL support cryo-survival and early embryonic development.

## Supporting information

S1 Table Baseline characteristics of patients who underwent embryo transfer using vitrified–warmed oocytes in the control and FA + MEL groups. BMI, body mass index; AMH, anti-Müllerian hormone; FSH, follicle-stimulating hormone; n, number; DOR, diminished ovarian reserve; PCOS, polycystic ovarian syndrome; SEM, standard error of the mean; FA, fatty acids; MEL, melatonin. Mixed factor refers to patients with ≥2 infertility factors (for example, male + uterine, or DOR + unexplained). Values are presented as the mean ± SEM.
(DOCX)

S2 Table Morphological grading of ICM and TE in blastocysts derived from vitrified–warmed oocytes in the control and FA + MEL groups. ICM, inner cell mass; TE, trophectoderm; n, number; N, total number of blastocysts assessed; FA, fatty acids; MEL, melatonin. n/N indicates the number of blastocysts assigned to each morphological grade per total number of blastocysts evaluated.
(DOCX)

## Abbreviations

IVF: in vitro fertilisation
ICSI: intracytoplasmic sperm injection
AMH: anti-Müllerian hormone
GnRH: gonadotropin-releasing hormone
ET: embryo transfer
FET: frozen-thawed embryo transfer
FA: fatty acids
MEL: melatonin
ATP: adenosine triphosphate
DMSO: dimethyl sulfoxide
HRT: hormone replacement therapy
ICM: inner cell mass
TE: trophectoderm
