# Supplementary material for: Fatty acid and melatonin-enriched warming: A novel approach using vitrified oocytes and early-stage embryos for patients with poor prognosis
Source: PLoS One. 2026 Apr 21;21(4):e0346886. doi: 10.1371/journal.pone.0346886 (PMC13098940; doi:10.1371/journal.pone.0346886)
Supplement: S1 Table — BMI, body mass index; AMH, anti-Müllerian hormone; FSH, follicle-stimulating hormone; n, number; DOR, diminished ovarian reserve; PCOS, polycystic ovarian syndrome; SEM, standard error of the mean; FA, fatty acids; MEL, melatonin. Mixed factor refers to patients with ≥2 infertility factors (for example, male + uterine, or DOR + unexplained). Values are presented as the mean ± SEM. (DOCX) [file pone.0346886.s001.docx]

**Supplementary Table 1. Baseline characteristics of patients who underwent embryo transfer using vitrified–warmed oocytes in the control and FA+MEL groups**

| Characteristics | Control  (n=62) | FA+MEL  (n=39) | *P*-Value |
| --- | --- | --- | --- |
|  |  |  |  |
| Age at oocyte vitrification (years) | 39.7 ± 0.52 | 38.6 ± 0.63 | 0.651 |
| Age at oocyte warming (years) | 41.3 ± 0.46 | 41.5 ± 0.61 | 0.745 |
| BMI (kg/m²) | 20.9 ± 0.25 | 21.1 ± 0.41 | 0.834 |
| Duration of infertility (years) | 2.7 ± 0.34 | 2.8 ± 0.76 | 0.624 |
| Number of previous IVF cycles | 3.4 ± 0.40 | 3.1 ± 0.46 | 0.869 |
| Number of previous embryo transfer cycles | 1.5 ± 0.23 | 1.4 ± 0.14 | 0.702 |
| Baseline FSH (mIU/mL) | 10.9 ± 0.79 | 13.5 ± 2.46 | 0.874 |
| AMH (ng/mL) | 2.6 ± 0.43 | 1.7 ± 0.53 | 0.074 |
| Endometrial thickness at transfer (mm) | 9.0 ± 0.23 | 8.0 ± 0.28 | 0.009 |
| Cause of infertility, | | | |
| Male factor | 12.9 (8/62) | 5.1 (2/39) | 0.203 |
| Uterine factor | 21.0 (13/62) | 17.9 (7/39) | 0.711 |
| Unexplained | 6.5 (4/62) | 7.7 (3/39) | 0.811 |
| DOR | 6.5 (4/62) | 10.3 (4/39) | 0.491 |
| PCOS | 9.7 (6/62) | 7.7 (3/39) | 0.733 |
| Mixed factor | 43.5 (27/62) | 51.3 (20/39) | 0.448 |

BMI, body mass index; AMH, anti-Müllerian hormone; FSH, follicle-stimulating hormone; n, number; DOR, diminished ovarian reserve; PCOS, polycystic ovarian syndrome; SEM, standard error of mean; FA, fatty acids; MEL, melatonin. Mixed factor refers to patients with ≥2 infertility factors (for example, male + uterine, or DOR + unexplained). Values are presented as the mean ± SEM.
