# Supplementary material for: Fatty acid and melatonin-enriched warming: A novel approach using vitrified oocytes and early-stage embryos for patients with poor prognosis
Source: PLoS One. 2026 Apr 21;21(4):e0346886. doi: 10.1371/journal.pone.0346886 (PMC13098940; doi:10.1371/journal.pone.0346886)
Supplement: S2 Table — ICM, inner cell mass; TE, trophectoderm; n, number; N, total number of blastocysts assessed; FA, fatty acids; MEL, melatonin. n/N indicates the number of blastocysts assigned to each morphological grade per total number of blastocysts evaluated. (DOCX) [file pone.0346886.s002.docx]

**Supplementary Table 2. Morphological grading of ICM and TE in blastocysts derived from vitrified–warmed oocytes in the control and FA+MEL groups**

|  |  |  |  |  |
| --- | --- | --- | --- | --- |
| Outcomes | | Control | FA+MEL | *P*-value |
| ICM morphology grade, n/N (%) | | | | |
| Grade A | | 1/40 (2.5) | 8/115(7.0) | 0.299 |
| Grade B | | 29/40 (72.5) | 73/115 (63.5) | 0.300 |
| Grade C | | 10/40 (25.0) | 34/115 (29.6) | 0.581 |
| TE morphology grade, n/N (%) | | | | |
| Grade A | | 1/40 (2.5) | 4/115 (3.5) | 0.763 |
| Grade B | | 12/40 (30.0) | 39/115 (33.9) | 0.650 |
| Grade C | | 27/40 (67.5) | 72/115 (62.6) | 0.579 |

ICM, inner cell mass; TE, trophectoderm; n, number; N, total number of blastocysts assessed; FA, fatty acids; MEL, melatonin. n/N indicates the number of blastocysts assigned to each morphological grade per total number of blastocysts evaluated.
